# Ca^2+^ toxicity and mitochondrial damage in acute pancreatitis: translational overview

**DOI:** 10.1098/rstb.2015.0425

**Published:** 2016-08-05

**Authors:** József Maléth, Péter Hegyi

**Affiliations:** 1 First Department of Medicine, University of Szeged, Szeged, Hungary; 2 MTA-SZTE Momentum Translational Gastroenterology Research Group, University of Szeged, Szeged, Hungary; 3 Institute for Translational Medicine, University of Pécs, Pécs, Hungary

**Keywords:** acute pancreatitis, mitochondrial damage, Ca^2+^ overload

## Abstract

Acute pancreatitis (AP) is a leading cause of hospitalization among non-malignant gastrointestinal disorders. The mortality of severe AP can reach 30–50%, which is most probably owing to the lack of specific treatment. Therefore, AP is a major healthcare problem, which urges researchers to identify novel drug targets. Studies from the last decades highlighted that the toxic cellular Ca^2+^ overload and mitochondrial damage are key pathogenic steps in the disease development affecting both acinar and ductal cell functions. Moreover, recent observations showed that modifying the cellular Ca^2+^ signalling might be beneficial in AP. The inhibition of Ca^2+^ release from the endoplasmic reticulum or the activity of plasma membrane Ca^2+^ influx channels decreased the severity of AP in experimental models. Similarly, inhibition of mitochondrial permeability transition pore (MPTP) opening also seems to improve the outcome of AP in *in vivo* animal models. At the moment MPTP blockers are under detailed clinical investigation to test whether interventions in MPTP openings and/or Ca^2+^ homeostasis of the cells can be specific targets in prevention or treatment of cell damage in AP.

This article is part of the themed issue ‘Evolution brings Ca^2+^ and ATP together to control life and death’.

## 1. Ca^2+^ is controlling secretory events in pancreatic acinar and ductal cells

Intracellular Ca^2+^ signalling plays central role in the regulation of the secretory processes of the exocrine pancreas. It is a well-known fact that in the exocrine pancreas acinar cells secrete digestive enzymes and pancreatic ductal epithelial cells secrete 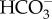 rich alkaline fluid that washes the digestive enzymes out from the pancreas. The prompt coordination of the secretory events of the two cell types is essential and Ca^2+^ has a central role in both pancreatic physiology and pathophysiology. Recent studies suggest that these two cell types cannot be handled separately since they are more likely integrated into a functional unit [1]. This is further amplified by the neurohormonal regulation of exocrine pancreatic secretion. It has been demonstrated that acetylcholine (the main stimulatory neurotransmitter of the pancreas) is released from the parasympathic nerve endings, releasing digestive enzymes from the acinar cells [2], whereas at the same time enhances the pancreatic ductal fluid and 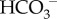 secretion via M_3_ metabotropic cholinerg receptor (M_3_R) mediated Ca^2+^ release [3]. In addition, the circulating hormone cholecystokinin (CCK) regulates pancreatic secretion via oscillatory Ca^2+^ signals [4]. In pancreatic ductal epithelial cells (PDECs), the role of CCK stimulation differs between species, in humans it has negligible direct effects, but remarkably potentiates the stimulatory effect of secretin on the 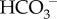 secretion [5]. The proper control of secretion is further potentiated by the strong synergy between Ca^2+^ and cAMP signalling [6]. The physiological roles of Ca^2+^ signalling in epithelial secretion have been outlined in more detail in excellent reviews [7–10].

## 2. The price of versatility: Ca^2+^ toxicity in acute pancreatitis

**Figure 1.**
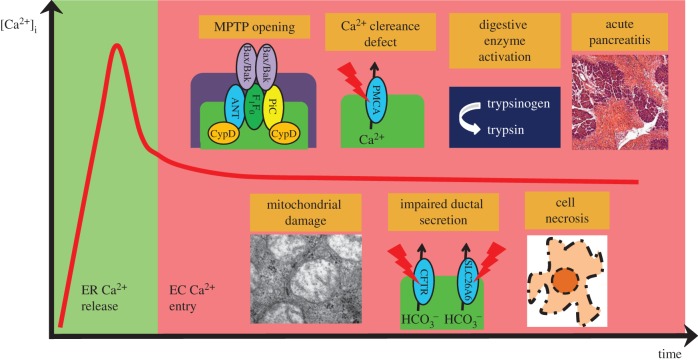
Hypothetical sequence of events in the pathogenesis of AP. Pancreatitis inducing toxic stress factors can release the intracellular Ca^2+^ from the stores, such as the endoplasmic reticulum (ER), or acidic organelles. However, the constant presence of toxins will lead to the elongation of the Ca^2+^ signals via multiple mechanisms. First, the ER Ca^2+^ depletion activates the influx of extracellular (EC) Ca^2+^. Second, the direct mitochondrial toxicity of the stress factors (such as bile acids or non-oxidative ethanol metabolites), increases reactive oxygen species production and the sustained Ca^2+^ increase will lead to the opening of the MPTP that will damage the mitochondria. The lack of intracellular ATP impairs the function of Ca^2+^ extrusion and reuptake pumps such as PMCA or SERCA. These changes together will generate a vicious cycle leading to inhibited secretion and intracellular activation of digestive enzymes in acinar cells and impaired ductal fluid and 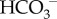 secretion. Altogether, these changes will trigger cell necrosis and AP.

Although it is well established that physiological Ca^2+^ signalling controls the normal pancreatic functions on multiple levels, it is also well documented that uncontrolled cellular Ca^2+^ overload leads to cellular damage and pathogenesis of acute pancreatitis (AP; figure 1). In this chapter, we will summarize the effects of the common stress factors that cause AP.

### (a) Bile acids

Biliary pancreatitis is one of the most common forms of AP, although the exact pathogenesis is not known in detail. One possible explanation is the ‘common channel’ theory, which suggests that an impacted gallstone creates communication behind the stone connecting the common bile duct to the pancreatic duct. This would theoretically allow bile acids (BAs) to reach the pancreatic ductal lumen or even the acinar cells [11]. However, this hypothesis was questioned by several studies suggesting that instead of the reflux mechanism, increased luminal pressure would cause the pancreatic damage [12–15]. Whether or not BAs reach the pancreatic tissue directly from the luminal side, several observations suggest that BA reaching the ductals cells from either basolateral or luminal sides can trigger multiple cellular responses in acinar and ductal cells that might contribute to the development of AP.

Earlier, our group showed that the hydrophobic, non-conjugated BA, chenodeoxycholate (CDCA) dose-dependently affects 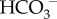 secretion of pancreatic ductal epithelia [16]. We found that lower concentration of CDCA (100 µM) stimulated and high concentration (1 mM) severely inhibited the ion transport activities including the ductal 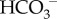 secretion. The explanation for this dual effect might be the type of Ca^2+^ signals triggered by CDCA. Luminal administration of 100 µM CDCA evoked short oscillatory Ca^2+^ signals, which were fully abolished by IP_3_ receptor inhibition. On the other hand, challenging the pancreatic ductal cells with 1 mM CDCA caused a sustained Ca^2+^ elevation [16] and severe damage of the mitochondrial morphology and function [17]. Interestingly, in our hands N,N′-[1,2-ethanediylbis(oxy-2,1-phenylene)]bis[N-[2-[(acetyloxy)methoxy]-2-oxoethyl]]-,bis[(acetyloxy)methyl]ester (BAPTA-AM) failed to prevent the mitochondrial damage and therefore the inhibitory effect of CDCA on the 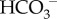 secretion [16], which might be explained by the existence of a Ca^2+^-independent direct mitochondrial toxicity of bile acids [18]. Similarly to ductal cells, pancreatic acinar cells respond with intracellular Ca^2+^ elevation to BA challenge [19] due to IP_3_R and ryanodine receptor activation. It is also well documented that taurolithocholicacid 3-sulfate diminishs cellular ATP production [20] and dissipate the mitochondrial membrane potential (Δ*Ψ*_m_), which was not affected by BAPTA-AM treatment [21]. Although BA directly affects the acinar cells, the observations of Perides *et al*. actually suggest that biliary pancreatitis is a receptor mediated disease [22]. They showed that the G-protein-coupled cell surface bile acid receptor (Gpbar1, or TGR5) is expressed at the apical membrane of pancreatic acinar cells and its activation is associated with pathological Ca^2+^ signals, intracellular activation of digestive enzymes and cell injury, i.e. the hallmarks of AP. Whereas the genetic deletion of Gpbar1 markedly reduced the severity of taurolithocholic acid 3-sulfate (TLCS)-induced, but not caerulein-induced AP. Very recently, Katona *et al*. provided solid evidence that specific BA might be used as treatment option against biliary pancreatitis [23]. They showed that pre-treatment of pancreatic ducts with ursodeoxycholate (UDCA) remarkably ameliorated the toxic effects of UDCA. Chenodeoxycholate-induced intracellular ATP depletion, mitochondrial injury, and as a consequence, cell death were completely prevented by UDCA, whereas the activity of the epithelial acid–base transporters was preserved in *in vitro* experiments. In addition, *in vivo* experiments showed that oral administration of UDCA significantly reduced the severity of CDCA-induced AP. Interestingly, UDCA had no effect on the sustained Ca^2+^ elevation triggered by CDCA, raising the possibility of a direct mitochondrial protective effect, which is yet to be determined. These observations nicely supplement the previous results of Seyhun *et al*., who showed that the endoplasmic reticulum (ER) chaperone tauroursodeoxycholic acid inhibits the unfolded protein response (UPR) *in vitro* [24] and *in vivo* [25]. This effect reduced the activation of UPR components and reduced intracellular trypsin activation, oedema formation and cell damage in pancreatic acinar cells.

### (b) Ethanol and non-oxidative ethanol metabolites

The second most frequent form of pancreatitis is alcohol-induced AP [26]. Whereas genetic factors seem to be involved in the disease development [27], several studies investigated the direct effects of ethanol and different ethanol metabolites on the exocrine pancreas. Ethanol and its oxidative metabolite acetaldehyde have moderate effects on the [Ca^2+^]_i_ in pancreatic acinar cells even in extremely high concentrations [28]. Whereas the non-oxidative ethanol metabolites (fatty acid ethyl esters, FAEE) induced sustained [Ca^2+^]_i_ elevation and a drop of cellular ATP leading to necrosis [28–30]. Importantly, the breakdown of FAEE to fatty acids (FA) by intracellular hydrolases significantly contribute to the toxic effects of non-oxidative ethanol metabolites [30]. This fact has been further emphasized in a recent elegant study by Huang *et al.* [31]. They showed that the inhibition of oxidative ethanol metabolism significantly enhance, whereas inhibition of non-oxidative ethanol metabolism augment pancreatic damage in an *in vivo* model of ethanol-fatty acid induced AP. On the other hand pancreatic ductal cells respond to low to high concentrations of alcohol, likewise to BA. Yamamoto *et al*. showed that 1 mM ethanol induces [Ca^2+^]_i_, elevation and augments fluid secretion, whereas high concentration moderately inhibits the stimulated fluid secretion in secretin-stimulated guinea pig pancreatic ducts [32]. Our group recently investigated the effects of ethanol and ethanol metabolites in more detail [33]. We showed that alcohol and fatty acids inhibit fluid and 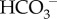 secretion, as well as cystic fibrosis transmembrane conductance regulator (CFTR) activity, in pancreatic ductal cells. Interestingly, in the case of FAEE only the inhibition of the CFTR channel was observed in high concentrations [34]; however, the inhibition of 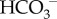 secretion was not observed [33]. The remarkable inhibitory effects of alcohol and fatty acids were mediated by sustained increase of intracellular Ca^2+^, inhibited adenosine 3’,5’-cyclic monophosphate and ATP production and depolarization of Δ*Ψ*_m_. We also showed that ethanol reduced expression of CFTR via multiple pathways, which in turn augmented the severity of experimental alcohol-induced AP in mice.

### (c) Other stress factors

As demonstrated above, the two most common pathogenic factors of AP—BA and ethanol—damage the exocrine pancreas via Ca^2+^ toxicity and mitochondrial injury. Notably, these cellular changes seem to be the key of AP pathogenesis since a considerable number of studies showed that other stress factors provoke the same alterations in Ca^2+^ signalling and energy metabolism. Intrapancreatic trypsinogen activation is a hallmark of AP pathogenesis and we showed earlier that trypsin acting via PAR2 on the luminal membrane induces intracellular Ca^2+^ elevation and inhibits the luminal acid/base transporters in PDEC [35]. Moreover, the inhibitory effect was abolished by BAPTA-AM preincubation, similarly to the inhibitory effects of ethanol and fatty acids. Very recently, Jin *et al*. investigated the pathomechanism of an iatrogen form of AP, the post-ERCP pancreatitis [36]. Using sophisticated *in vitro* and *in vivo* models, they showed that exposure of pancreatic acinar cells to iohexol (a radiocontrast agent) triggered sustained intracellular Ca^2+^ elevation. The downstream activation of NF-κB and NFAT is completely abolished by the suppression of the Ca^2+^ signals. Moreover, they proved that the downstream effects of Ca^2+^ were mediated by calcineurin since genetic, or pharmacological inhibition of calcineurin prevented the radiocontrast-induced damage. This interesting study further underlines the central role of pathophysiological Ca^2+^ signalling in the pathogenesis of AP regardless of the etiological factor.

## 3. Sources of Ca^2+^ in pancreatic acinar and ductal cells

### (a) Ca^2+^ release from the endoplasmic reticulum

Agonist binding (Ach, ATP) to G-protein-coupled receptors activate phospholipase C β (PLCβ) in pancreatic acinar and ductal cells. The activated PLCβ releases inositol trisphosphate (IP_3_) by hydrolysing phosphatidylinositol 4,5-bisphosphate (PIP_2_) [37]. Under physiological conditions, the intracellular Ca^2+^ signals have a strict spatio-temporal localization [38,39], mostly limiting Ca^2+^ signals to the apical pole of the cells. As in other non-excitable cell types, this is ensured by two ATP-dependent pumps that clear the cytosol from the free Ca^2+^. The sarco/endoplasmic reticulum Ca^2+^-ATPase (SERCA) pumps and the plasma membrane Ca^2+^-ATPase (PMCA) pumps move Ca^2+^ from the cytosol to the ER and the extracellular space, respectively. This activity restores basal intracellular Ca^2+^ levels and refills the ER Ca^2+^ stores. In PDEC, the Ca^2+^ signalling is not characterized in such detail; however, the overall polarity of the ductal cells including the ion channels and transporters, IP_3_ receptors and mitochondria [17], suggest a very similarly regulated Ca^2+^ signalling, like in acinar cells. Further studies are required for the clarification of these questions.

### (b) Extracellular Ca^2+^ influx

The complex role of extracellular Ca^2+^ influx to orchestrate non-excitable cell functions has been established several decades ago [40]; however, the molecular components participating in the process remained unknown until 2005. Hoth *et al*. found that agonist-mediated depletion of the intracellular Ca^2+^ stores induced a Ca^2+^ selective sustained inwardly rectifying current, which was termed I_CRAC_ (calcium release-activated calcium current) [41]. The real revolution of the field began by the discovery of the ER Ca^2+^ sensor stromal interaction molecule 1 (Stim1) [42] and the plasma membrane Ca^2+^ channel Orai1 [43,44]. Briefly, the process of store operated Ca^2+^ entry (SOCE) consist of the following elements. In resting conditions the ER Ca^2+^ stores are refilled and Stim1 distributes in the ER membrane. However during physiological stimulation the ER Ca^2+^ stores are quickly depleted, which induces the dissociation of the bound Ca^2+^ from the EF hand of Stim1. This is followed by a conformational change and translocation of Stim1 to defined ER-PM junctions, termed as puncta formation [45]. This translocation is required for the activation of the plasma membrane Ca^2+^ influx channel Orai1, where the Stim Orai1-activating region (SOAR) and polybasic domains of Stim1 interact with different binding sites of Orai1 that results in clustering and activation of the channel [46]. In addition to Orai1, other possible Ca^2+^ entry channels that seem to play a role in Stim1-mediated SOCE are the TRPC channels [47,48]. These channels function as Ca^2+^-permeable non-selective cation channels mediating receptor evoked Ca^2+^ influx in many cells [49]. SOCE have been investigated mostly in acinar cells of various exocrine glands, as models of polarized epithelial cells [50–52]. Interestingly, the role of SOCE in the physiological functions of PDEC, especially in 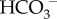 secretion remained elusive. Kim *et al*. found that intracellular Ca^2+^ elevation, caused by the activation of SOCE might play a role in exocytosis in pancreatic ductal cells isolated from dog main pancreatic duct [53,54]; however, they did not investigate 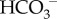 secretion of PDEC, which therefore needs further investigation.

## 4. Mitochondrial Ca^2+^ handling and Ca^2+^ overload of mitochondria

During physiological Ca^2+^ signalling, mitochondria takes up Ca^2+^, which has been shown to directly increase energy output by enhancing the activity of tricarboxylic acid cycle dehydrogenases and the ATP synthase [55]. The pioneer work of Rizzuto *et al*. highlighted that the cytosolic Ca^2+^ signals propagate to the mitochondria [56] and a couple of years later Csordas *et al*. found that ER membrane and the outer mitochondrial membrane form a quasi-synaptic connection [57] that is the structural bases of the Ca^2+^ hotspots [58]. Despite the functional characterization of the mitochondrial Ca^2+^ signalling the molecular background of the process was not known. In 2011, two groups independently identified the mitochondrial Ca^2+^ uniporter (MCU), an inner mitochondrial membrane protein that is responsible for the mitochondrial Ca^2+^ uptake [59,60]. The Ca^2+^ efflux from the mitochondria is mediated by the mitochondrial Na^+^/Ca^2+^ exchanger (NCLX) [61], thus the mitochondrial Ca^2+^ level is tightly regulated under physiological conditions. However, pathophysiological signals can lead mitochondrial injury, which can activate both apoptosis and necrosis. The classical mitochondrial apoptotic pathway involves the outer membrane permeabilization by Bax and Bak (two members of the pro-death Bcl-2 family) that will allow apoptotic factors like cytochrome *c*, Smac/DIABLO and apoptosis inducing factor to be released from the intermembrane space into the cytosol, leading to cell death by apoptosis [62]. On the other hand, Ca^2+^ overload or increased reactive oxygen species (ROS) production can cause the opening of mitochondrial permeability transition pore (MPTP) that results in the loss of mitochondrial inner membrane potential, uncoupling of the respiratory chain with a consequent drop of mitochondrial ATP synthesis, and increased permeability of the inner mitochondrial membrane that eventually leads to mitochondrial swelling, rupture and necrotic cell death [63,64]. Notably, recent studies lead to the reconsideration of the role of MPTP in cellular physiology, since it has been proved to be important in several physiological processes such as energy metabolism [65], mitochondrial Ca^2+^ efflux [66] and ROS signalling [67] as well. The molecular identity of MPTP is still a matter of investigation [68]. The historical model of MPTP included the voltage-dependent anion channel (VDAC) in the outer mitochondrial membrane, the adenine nucleotide translocator (ANT) in the inner mitochondrial membrane, and CypD as its regulator in the matrix of the mitochondria [69]. However recent intensive efforts revealed new molecules that might contribute to the MPTP formation (reviewed in detail [68,70]). A growing number of evidence suggest that VDAC is not very likely to contribute to the MPTP formation. On the other hand, studies on ANT suggest that it is not required for MPTP formation, but it regulates MPTP activity [71]. CypD is an important regulator of MPTP as supported by genetically modified mice [72] and pharmacologic inhibition of CypD by cyclosporine A [73]. On the other hand, several studies suggested that the activity of F_1_F_0_ ATP synthase or the proapoptotic Bax/Bak proteins [74] are required for proper MPTP function [75], whereas other proteins, such as mitochondrial phosphate carrier, might impact the pore opening indirectly [76]. At the moment the role of MPTP in the pathogenesis of AP is supported by limited, but still solid evidence. Mukherjee *et al*. demonstrated that both genetic and pharmacologic inhibition of MPTP opening (using the Cyclophilin D-deficient *Ppif* gene knockout mice, or *in vivo* treatment with cyclosporine A derivates, respectively) significantly ameliorated pancreatic damage in different experimental AP models in mice [77]. Importantly MPTP blockade protected the pancreatic acinar cells from necrosis whereas apoptosis was not affected, which is in strong agreement of earlier studies [72].

## 5. Novel therapeutic targets in acute pancreatitis

**Figure 2.**
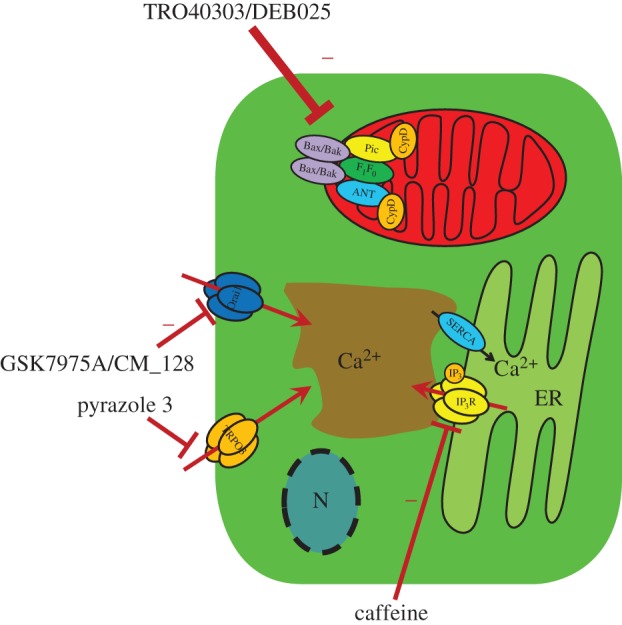
Novel therapeutic targets in AP. Experimental studies from recent years identified several proteins in cellular Ca^2+^ signaling machinery that might be potential target molecules in AP treatment. Caffeine and dimethylxanthines were shown to block IP_3_-mediated Ca^2+^ release from the ER that decreased the severity of AP in experimental models. Similarly, the inhibition of the plasma membrane Ca^2+^ influx channels Orai1 and TRPC3 reduced the severity of AP in animal models. Another treatment possibility might be the inhibition of the MPTP opening, which improved the disease outcome in rodents.

In pancreatic acinar cells, IP_3_-mediated Ca^2+^ release from the ER is an essential component of the physiological response to agonist stimulation, but it could also contribute to the pathological Ca^2+^ overload of the cells evoked by toxic factors that induce AP (cerluien hyperstimulation, bile acids, or ethanol and ethanol metabolites) [39]. Caffeine is a known inhibitor of IP_3_Rs due to multiple actions that include the inhibition of phospholipase C-mediated production of IP_3_ [78], antagonism of IP_3_Rs [79] and direct binding to IP_3_Rs that reduce the channels open-state probability [80]. Interestingly, coffee consumption moderately reduces the risk of alcohol-associated pancreatitis suggesting that the inhibitory effect of caffeine on IP_3_-mediated Ca^2+^ signalling may be protective in AP [81]. Based on these considerations Huang *et al*. recently studied the effects of caffeine and its xanthine metabolites on pancreatic acinar IP_3_R-mediated Ca^2+^ signalling and experimental AP [82]. They found that caffeine and dimethylxanthines (but not monomethylxanthines) blocks IP_3_-mediated Ca^2+^ oscillations in response to uncaged IP_3_ or toxins, prevented mitochondrial depolarization and necrotic cell death *in vitro* and significantly impaired the severity of experimental AP in three different models. These observations suggest that caffeine, or its metabolites might be suitable starting points to develop therapy for AP (figure 2).

As discussed above, store operated Ca^2+^ entry could be a key component in the development of cellular Ca^2+^ overload. Earlier Kim *et al*. showed that genetic [83] or pharmacological inhibition (using the TRPC3-specific inhibitor pyrazole 3) [84] of TRPC3 significantly reduce the sustained Ca^2+^ elevation in pancreatic acinar cells evoked by cell stressors (bile acid or fatty acid ethyl ester). In addition, it prevented the pathological inhibition of digestive enzyme secretion and markedly reduced intracellular trypsin activation and excessive actin depolymerization *in vitro* and the severity of pancreatitis *in vivo*. Recently, Gerasimenko *et al*. demonstrated the pharmacological inhibition of another Ca^2+^ entry channel Orai1 by a specific inhibitor called GSK-7975A which prevents acinar cell necrosis *in vitro* [85]. This important observation was supported by Wen *et al*., who tested the effects of two specific Orai1 inhibitors (GSK-7975A and CM_128) in isolated human and rodent pancreatic acinar cells and in different experimental AP models [86]. They showed that both Orai1 inhibitors prevented the sustained Ca^2+^ elevation *in vitro* and significantly impaired signs of pancreatic injury including pancreatic oedema, inflammation and necrosis in all tested experimental models.

Mitochondrial permeability transition is a key feature of cellular damage in many cell types and diseases (see above); therefore, MPTP blockers are under detailed clinical investigation in different studies. In a recent clinical study, the efficacy and safety of TRO40303 (an MPTP inhibitor) have been evaluated for the reduction of reperfusion injury in patients undergoing revascularization for ST-elevation myocardial infarction (MITOCARE study) [87]. This study did not show any effect of TRO40303 in limiting reperfusion injury of the ischaemic myocardium. In another recently completed CIRCUS trial, the effects of i.v. administrated cyclosporine have been evaluated on the clinical outcome of patients with anterior STEMI [88]. Similarly to the MITOCARE study, CIRCUS trial did not report any improvement in the cyclosporine-treated patients. The reasons for the failure of the studies might be explained by pharamological limitations of the administrated compounds [89] that include low tissue penetration due to the lack of collateral blood flow and high metabolism of the compound in the blood. In addition, MPTP blockers have been suggested to be beneficial in hepatitis C therapy, since they inhibited hepatitis C virus (HCV) replication by preventing a cyclophilin-A induced cis–trans isomerization in domain II of NS5A [90]. However, it was not investigated in clinical trials further. Very recently, Mukherjee *et al*. tested the effect of MPTP inhibition on the severity of AP in rodent experimental AP models [77]. They have shown that the inhibition of MPTP with pharmacological compounds (two cyclosporine A derivate: DEB025 or TRO40303), or genetic deletion of the *Ppif* gene (that encodes cyclophylin D, a component of MPTP) significantly decreases the severity of AP in different independent models. These observations suggest that the MPTP inhibition might be potentially beneficial in the AP therapy. Other indirect evidence for this hypothesis has been provided by Judak *et al*., who showed that the supplementation of cellular ATP *in vitro* diminished the inhibitory effect of ethanol metabolites on the ion transport activities in isolated guinea pig pancreatic ductal cells [34]. These results suggest that the restoration of the cellular energy level can be beneficial in AP, which can prevent the cellular dysfunction and cell damage.

## 6. Closing remarks

Although there are several promising results and potential drug targets that play a role in the pathogenesis of AP, it remains a great challenge for researchers and clinicians. A number of unanswered questions are waiting for answers. Moreover, it will take several years to test the experimental results on clinical patients as well. To be able take up these challenges, clinicians and researchers should work closely together in the future.
